# Mechanism of (−)-epigallocatechin gallate (EGCG) dimerization by low-temperature plasma

**DOI:** 10.1038/s41598-022-19806-0

**Published:** 2022-09-13

**Authors:** Seungil Park, Gyeong Han Jeong, Sung Hoon Jee, Tae Hoon Kim, Seong Bong Kim

**Affiliations:** 1 Institute of Plasma Technology, Korea Institute of Fusion Energy, Gunsan, Republic of Korea; 2 Department of Food Science and Biotechnology, Daegu University, Gyeongsan, Republic of Korea

**Keywords:** Biochemistry, Physics

## Abstract

The efficient dimerization of (−)-Epigallocatechin gallate (EGCG), which is the major bioactive constituent isolated from the leaves of *Camellia sinensis*, was initially reported without changes in its stereochemistry using low-temperature plasma. The contribution of plasma during the dimerization of EGCG in a methanolic solution was quantified using a major factor, with the major factor demonstrated based on the contents of newly generated products, in this case the sum of oolonghomobisflavans A and B depending on the plasma treatment method. Samples were treated in three methods: plasma direct treatment, an indirect treatment using only reactive species, and an indirect treatment using effects other than those by reactive species. Ozone was identified as a major factor during the plasma treatment, and the operating ranges of the ozone concentration for regulated dimerization were evaluated. The mechanism by which EGCG synthesizes dimers A and B during the treatment process using low-temperature plasma was investigated using the derived major factor and prior literature. The ozone generated by the plasma reacted with methanol to form formaldehyde, and dimers A and B were synthesized by oligomers through a methylene-bridge by the formaldehyde. A plausible pathway of regulated dimerization was deduced based on these results, and the mechanism of EGCG dimerization by plasma is described using this pathway.

## Introduction

Natural polyphenols have attracted great interest worldwide due to their health-promoting properties, and is present as major component in a various foods and in medicinal plants^1^. Polyphenols containing at least two phenolic functional groups reportedly have the potential to improve human health owing to their significant antioxidant properties as anti-aging medications, for cancer prevention, and to treat cardiovascular disorders^2^. However, several polyphenols have limited clinical efficacy due to instability under oxidative and physiological conditions^3, 4^. Thus, structural modification studies of natural polyphenols using enzymatic and microbial modifications are being conducted to synthesize valuable new derivatives with improved biological efficacy. (−)-Epigallocatechin gallate (EGCG) is only one of commonly consumed natural polyphenols found in the leaves of green tea (*Camellia sinesis*) that has been studied to have a widely range of pharmacological capacities, such as anti-angiogenic, anti-diabetic, hypocholesterolemic, anti-bacterial, and anti-aging effects^5–12^. Recent, EGCG has been reported to have potent anti-cancer effects in vitro and in vivo^13–15^, and is also used as an effective drug delivery system^16^. However, EGCG is quite unstable under oxidative conditions and the low bioavailability. Several studies have examined the effects of molecular modifications and improvement on the biological activities of EGCG using various chemical change methods, such as peroxyl radical reaction and enzymatic oxidation processes^17, 18^. Additionally, plasma has been proposed as a viable method for the structural modification and synthesis of natural tea catechin^19–21^.

Plasma technology has been widely used in various fields. Examples include plasma processing in the semiconductor and display industries, surface modification, nanoparticle synthesis, gasification, catalysis, and even sterilization in the distant past. Moreover, many types of plasma apparatuses have been studied in relation to these applications. Low-temperature plasmas that can be generated at atmospheric pressure and at temperatures below 40 °C open up a range of biomedical and agricultural applications. Dielectric Barrier Discharge (DBD) is a widely used low-temperature atmospheric pressure plasma that has various effects on the target depending on the operating parameters. Under plasma treatment, sample can be effected by one of factors which are reactive species, UV radiation, heat, electric fields, and charged particles or by a synergistic combination of these factors. Reactive species, which is a major factor in atmospheric pressure plasma, such as ozone (O_3_) and nitrogen oxides (NO_x_), can have a chemical effect on the samples. Photons (UV/VIS), heat, electric fields, and charged particles (electrons, ions) can all have physical effects on the target during a plasma treatment.

Because EGCG is quite unstable under oxidative conditions, numerous studies focusing on structural modifications and enhancements of the biological properties of EGCG have been carried out. In our previous work^19^, it was demonstrated that new compounds were generated when the sample was directly irradiated with DBD plasma. These newly generated products were identified as oolonghomobisflavans A and B. This result suggests that such a plasma treatment would be an effective biotechnology by which to realize structural modifications with improved bioactivity. However, researchers thus far have not examined the effects of plasma on the regulated dimerization of EGCG in a methanolic solution. In this paper, the major factor and operating conditions of a plasma treatment are investigated through changes in plasma treatment methods, and a mechanism that utilizes a plausible pathway of regulated dimerization by a plasma treatment is proposed based on the outcomes here and in earlier work.

## Methods

### Plasma apparatus and experimental setup

**Figure 1:**
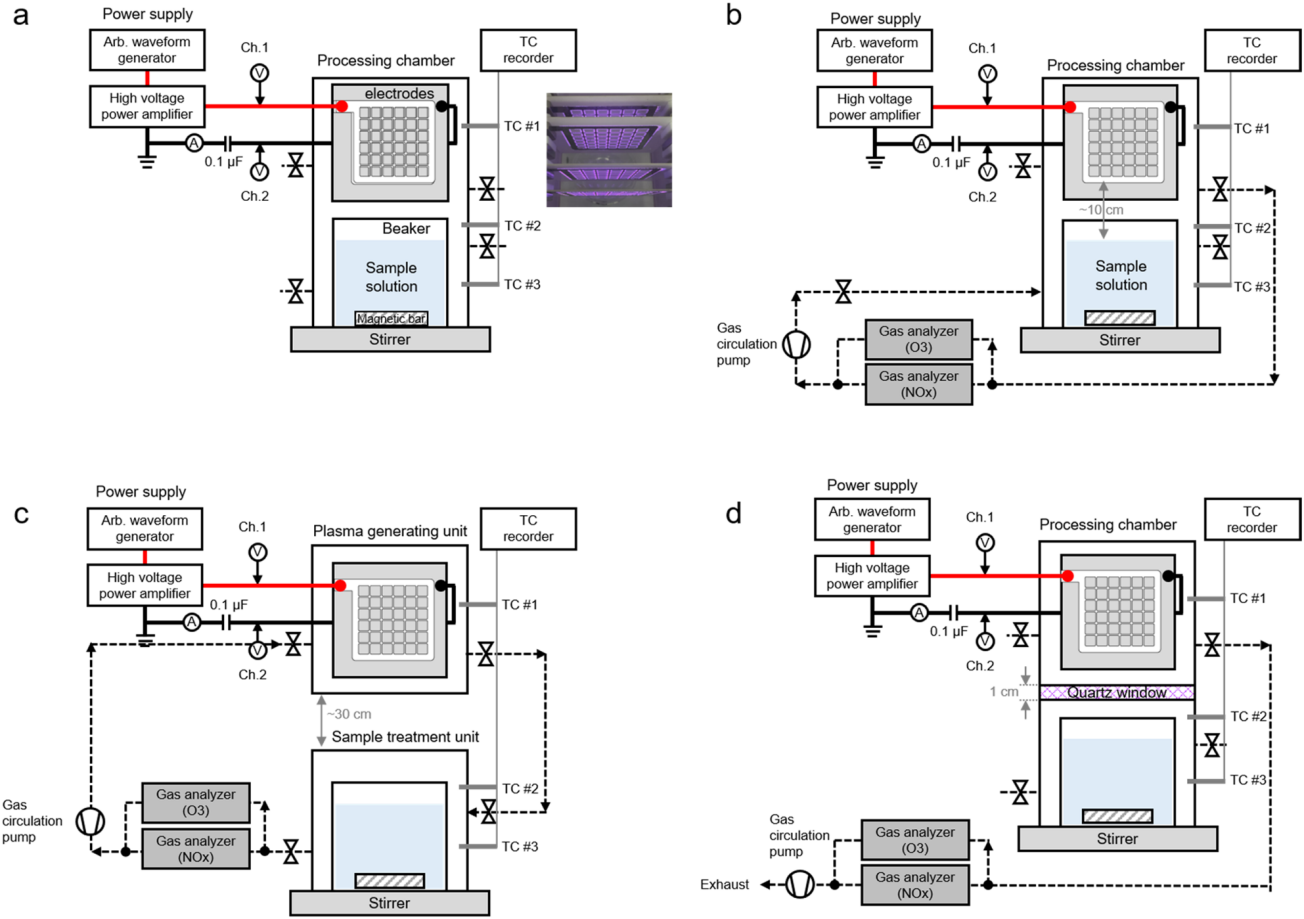
Schematic drawing of the test setup for the plasma treatment: (**a**) direct plasma treatment without gas circulation, (**b**) direct plasma treatment with gas circulation, (**c**) indirect plasma treatment to confirm the effect of the plasma reactive species with gas circulation, and (**d**) indirect plasma treatment to confirm the effect without the plasma reactive species and with gas circulation.

Figure 1 shows a schematic drawing of the test setup for the plasma treatment. The treatment apparatus consists of four surface dielectric barrier discharge (SDBD) electrodes, a processing chamber, and a power supply. One electrode consists of two metal sheets attached to both sides of a 100 × 100 mm^2^ alumina plate with a thickness of 0.7 mm. One metal sheet has a 6 × 6 rounded square open pattern, and the other has no open area. Both sheets are composed of a nickel-chromium alloy to prevent oxidation by the highly reactive oxidizing species used during the plasma operation. The processing chamber, made of Teflon to ensure low chemical reactivity, is divided into a plasma-generating unit and a sample treatment unit, and the two units are fastened using a lockable toggle latch and an O-ring for a gas-tight seal. High voltage was generated using an arbitrary waveform generator (Tektronix AFG3021C) and a high-voltage power amplifier (Trek 5/80) and was applied parallel to the four SDBD electrodes. The power applied to the plasma electrodes was monitored using a 1000:1 high-voltage probe (Tektronix P6015A), a current probe (Tektronix P6021A), a 100 nF capacitor, and an oscilloscope (Tektronix MDO 4024C), as shown in Fig. 1. To measure the gaseous reactive species generated by the plasma, a gas analyzer and gas circulation configuration were also included here as opposed to an earlier test setup. The gas in the plasma-processing chamber was supplied to the gas analyzer using a gas circulation pump operating at 10 *lpm*, and the measured gas was supplied back to the processing chamber through an exhaust line. The concentrations of the reactive species were observed using an O_3_ gas analyzer (Anseros GM-PRO), a NO_x_ gas analyzer (EcoPhysics CLD 60), and a gas circulation pump. Given that the measured concentrations change over time, the concentration in each case was calculated by dividing the area of the measured value over time by the discharge time. Because natural compounds such as EGCG are sensitive to the processing temperature, the gas temperatures inside the processing chamber were monitored using thermocouples installed at the location indicated in Fig. 1.

### Plasma treatment method

Low-temperature plasma generated using ambient air at atmospheric pressure produces several factors, such as reactive neutral species (reactive oxygen and nitrogen species), charged particles (electrons, ions), UV radiation, and heat, and an electric fields is generated by the voltage applied to the electrode for discharge, with the range of influence of each factor depends on the operating parameters. In this work, we performed measurements in the same way after establishing identical operating conditions, specifically applied voltage and frequency, discharge gas, volume and concentration of sample, and stirring speed, except during the plasma treatment method. The effectiveness of a plasma treatment for the regulated dimerization of EGCG was demonstrated by identifying the major factors according to the three treatment methods, which were a direct treatment, an indirect treatment using only reactive species, and an indirect treatment excluding reactive species. First, the direct plasma treatment directly generates plasma approximately 10 cm above the sample in the processing chamber and processes the sample using the effects of all factors generated during the discharge step, as shown in Fig. 1b. Second, the indirect plasma treatment using only reactive species treats the sample using only gaseous reactive oxygen and nitrogen species (RONS), the most important factors in atmospheric pressure plasma, as shown in Fig. 1c. The processing chamber is separated into a plasma-generating unit and a sample-processing unit using a windowless cover, and the distance between the two units was approximately 30 cm to minimize effects other than those by the RONS. Lastly, the indirect plasma treatment excluding reactive species uses the effects of remaining factors, in this case the UV radiation, heat, and electric field factors while excluding the RONS effects. Because the plasma treatment is conducted with the sample placed at least 10 cm away from the plasma, the effects of charged particles can be disregarded. A partition wall with a thickness of 1 cm and with a quartz window for transmitting light, heat, and the electric field was installed between the plasma-generating unit and the sample-processing unit in the processing chamber. The gas in the plasma-generating unit was exhausted through a gas analyzer using a circulation pump, and the sample-processing unit blocked the supply and exhaust of external gas by means of a gas valve as shown in Fig. 1d.

### Sample preparation and operation condition

**Figure 2:**
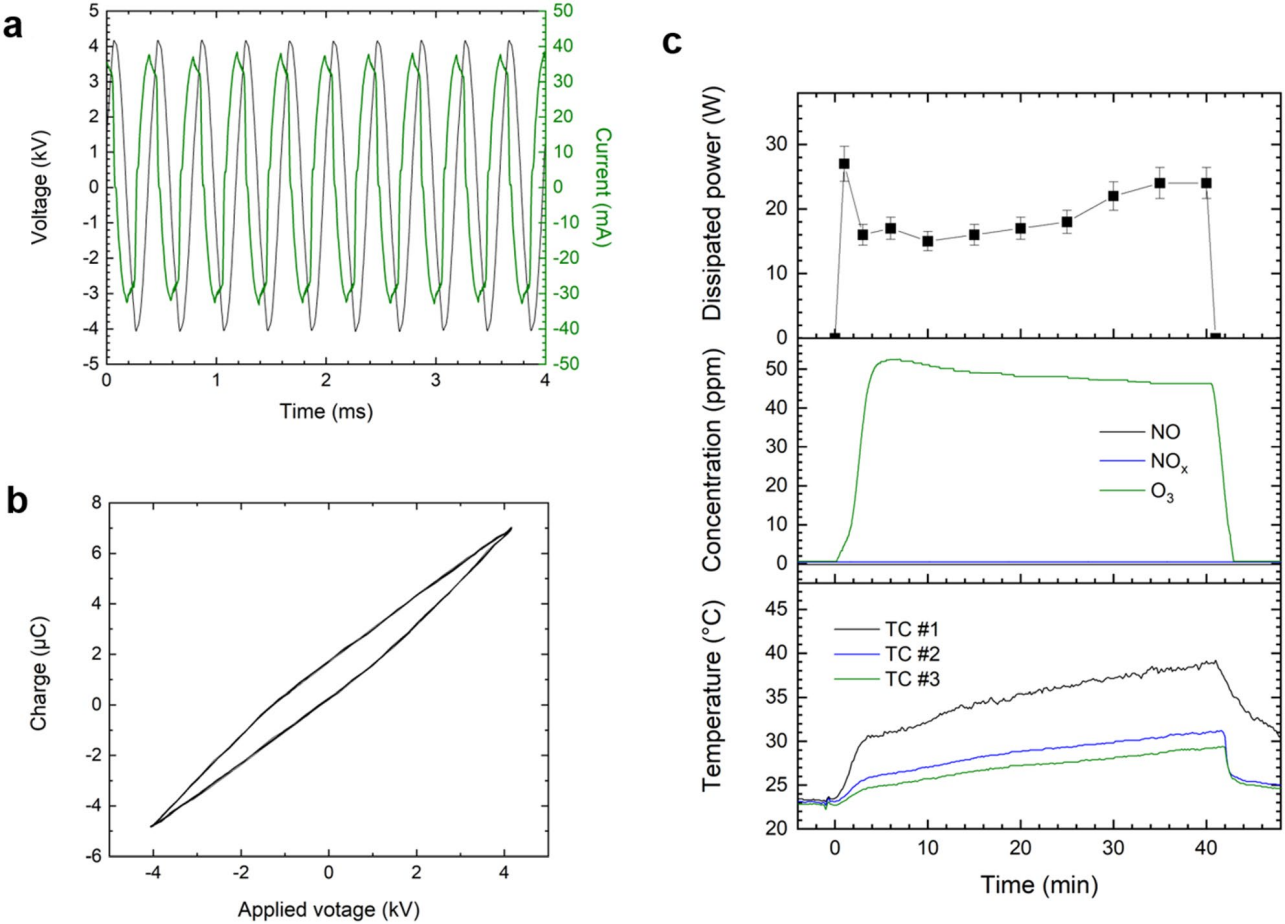
Characteristics of the plasma apparatus based on a SDBD electrode: (**a**) Voltage and current waveform applied to four SDBD electrodes, (**b**) Lissajous figure, and (**c**) time evolution of the dissipation power, concentrations of NO_x_ and O_3_ generated by the plasma, and gas temperatures inside the processing chamber.

Samples were created by dissolving EGCG (500 mg) in a methanolic solution (2.5 l). The reagents (−)-epigallocatechin gallate, methanol, formic acid (HPLC grade), ethyl acetate, and deuterated methanol used for the preparation and analysis of the samples were purchased from Sigma–Aldrich (St. Louis, MO, USA). The samples were irradiated with plasma for 10, 40, and 60 min to verify the effects of gas circulation upon the installation of the gas analyzer while stirring with a magnetic bar at 300 rpm, after which they were treated to identify the major factors in the plasma treatment using the three test setups. During the operation, plasma was generated at both sides of the SDBD electrodes using ambient air (temperature of 22.6 °C and 61% relative humidity) and a peak-to-peak sine wave at about 8 kV with a driving frequency of 2.5 kHz. The electric power dissipated to the plasma during the operation was calculated and found to be approximately 20 W using the Lissajous method. Figure 2 shows the voltages and current applied to the four SDBD electrodes as well as the Lissajous figure, time evolution of the dissipation power, the concentrations of NO_x_ and O_3_ generated by the plasma, and the temperature changes inside the chamber over a time of 40 min.

### Sample analysis

**Figure 3:**
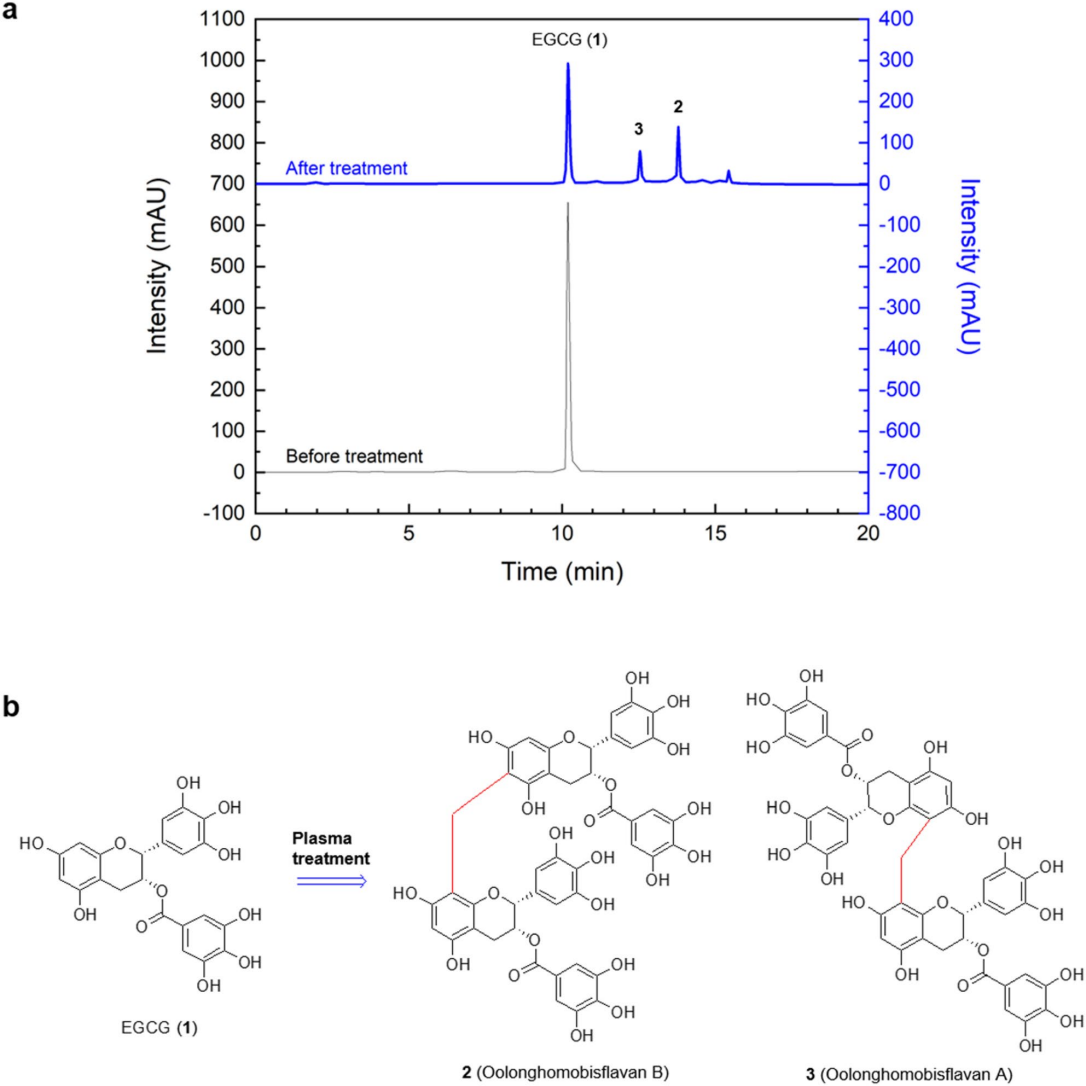
(**a**) HPLC chromatograms of samples before and after a plasma treatment for 40 min, and (**b**) Chemical structures of new products generated from EGCG by the plasma treatment.

After the plasma treatment, the solution was evaporated to remove the solvent promptly. The evaporated sample was analyzed after it was dissolving in a solvent of 0.1% formic acid and acetonitrile for an high-performance liquid chromatography (HPLC) analysis in order to confirm the compound newly generated by the plasma treatment. Figure 3 shows HPLC chromatograms of the samples before and after a 40 min plasma treatment and chemical structures of new compounds generated from EGCG by the plasma treatment. The treated samples were isolated, identified, and quantified according to the procedure explained below to evaluate the effect of the plasma treatment. First, the dried reactants after evaporation were suspended in 10% H_2_O in MeOH (100 mL), after which the solutions were partitioned with ethyl acetate (EtOAc, 3 × 100 mL) to yield a dried EtOAc-soluble layer (350 mg). A portion of the EtOAc-soluble layer was directly passed through a column chromatograph (YMC GEL ODS AQ 120-50S, 1 cm i.d. × 39 cm, particle size 50 μm; YMC Co., Kyoto, Japan) and was then eluted with aqueous MeOH, to yield pure compounds **2** (42.3 mg, *t*_R_ 13.8 min) and **3** (12.6 mg, *t*_R_ 12.5 min). Subsequently, a comparative analysis of spectroscopic data, in this case 1D-, 2D-NMR, and FABMS, along with reference data was conducted to identify the structure of the isolated pure compounds. Finally, a chromatographic analysis was performed via HPLC (LC-20AD, Shimadzu, Tokyo, Japan) with a photodiode-array detector (SPD-M20A, UV 280 nm; Shimadzu, Tokyo, Japan) for a quantitative analysis of the compounds newly generated from EGCG as induced by the plasma treatment. A column packed with a filler (YMC-Pack ODS A-302, 4.6 mm i.d. × 150 mm, particle size 5 μm; YMC Co., Kyoto, Japan) and solvents consisting of a gradient mode with an initial amount of 5% CH_3_CN in 0.1% HCOOH/H_2_O that increased to 100% CH_3_CN over 20 min (flow rate: 1.0 mL/min; at 40 °C) were used for the analysis. Details pertaining to the processing and analysis of the concentrated samples after the plasma treatment are available in the literature^19^.

## Results and discussion

### Sample treatment conditions

**Figure 4:**
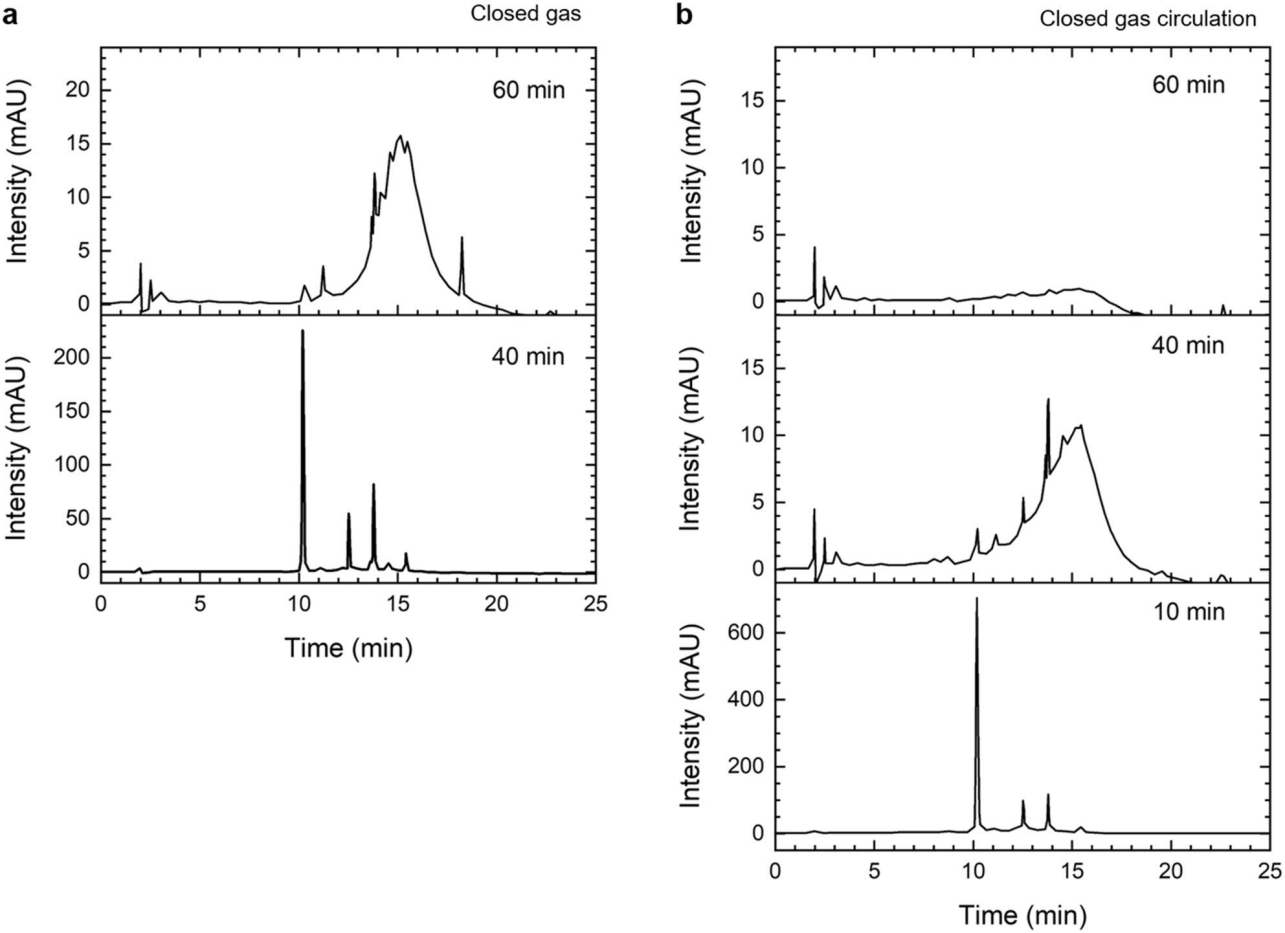
HPLC chromatograms of samples after plasma treatment for 60, 40, and 10 min: (**a**) Closed gas, and (**b**) Closed gas circulation.

The major factor contributing the most to regulated dimerization among the factors generated during the discharge step was identified based on the content of the newly generated products which were identified to be oolonghomobisflavans B (2) and A (3) in previous studies^19^. Before examining the influence of the plasma treatment, we confirmed whether the gas circulation caused by the addition of the gas analyzer changed the plasma treatment conditions. Figure 4 shows HPLC chromatograms of the sample components according to the plasma treatment time under identical operating conditions, except for the circulation of the gas. Sample ingredients generated by the plasma treatment show differences depending on whether or not gas was circulated at the same treatment time. The sample generated dimeric EGCG analogues 2 and 3 at the appropriate plasma treatment time (40 min in closed gas, 10 min under closed gas circulation) but formed an oligomer (oligomerization) as the treatment time was increased (60 min in closed gas, 40 min with closed gas circulation), eventually degrading (60 min in closed gas circulation) as shown in Fig. 4. Thereafter, the plasma treatment time was held constant at 10 min under closed gas circulation conditions (Supplementry Fig. 1).

### Major factor of plasma treatment

**Figure 5:**
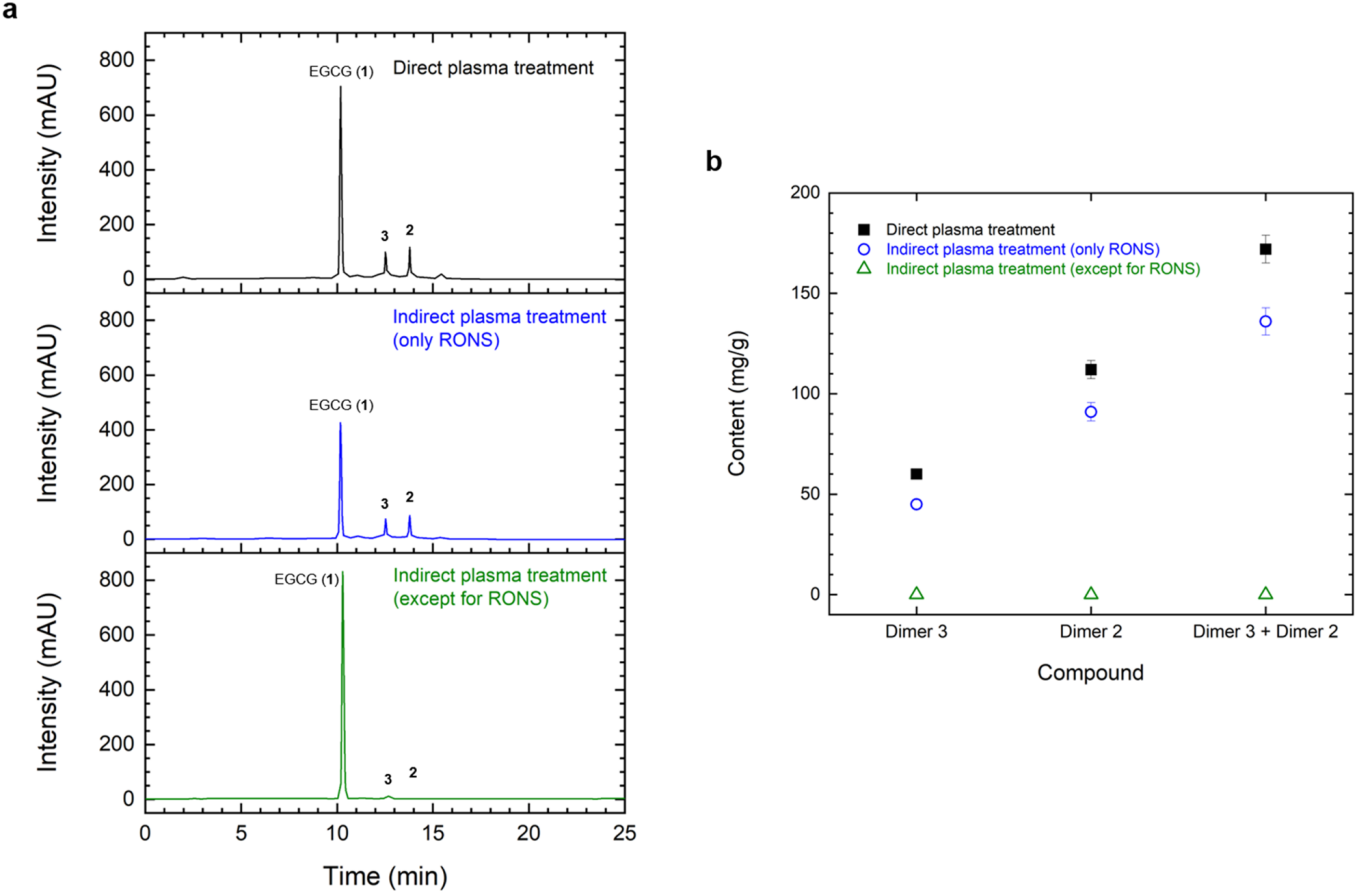
(**a**) HPLC chromatograms of samples according to the plasma treatment conditions, and (**b**) contents of individual components generated by the plasma treatment.

Figure 5 shows HPLC chromatograms of the treated sample and the content (sum of dimers 2 and 3) of the newly formed compounds produced by the direct plasma treatment, the indirect plasma treatment using only RONS, and the indirect plasma treatment without RONS under identical operating conditions. When the sample was directly treated with plasma, the amount of newly generated compounds produced was highest at about 172 mg/g. When the sample was treated with only RONS, the newly formed compound amounted to approximately 136 mg/g. No major compound was produced when the samples were treated while excluding RONS. The ozone concentrations according to the plasma treatment method were measured and found to be 39 ppm, 30 ppm, and 307 ppm, respectively. However, the concentrations of NO and NO_x_ did not change. During the discharge step, the maximum gas temperatures of TC #3 located next to the sample were 25.3 °C, 22.8 °C, and 23.7 °C, respectively, and the measured power consumption in all three cases was approximately 20 W. The major factors of the plasma treatment for regulated dimerization were identified as reactive oxygen and nitrogen species, especially ozone.

### Effects of plasma on dimerization

Although it was confirmed that ozone was the main factor during the plasma treatment upon a change of the plasma treatment method, a difference of about 20% was observed between the compounds produced by the direct plasma treatment and indirect treatment using only ozone. In order to understand the difference according to the plasma treatment method, the samples were exposed to conditions in which effects other than those by the reactive species were enhanced by increasing the voltage applied to the electrode. A sine waveform with a peak-to-peak amplitude of approximately 9 kV and frequency of 2.5 kHz was applied to the electrodes and the dissipated power was calculated and found to be about 26 W. The measured ozone concentrations according to the plasma treatment method were 52 ppm, 92 ppm, and 645 ppm, respectively, and the concentrations of NO and NO_x_ did not change. The maximum temperatures of TC #3 located next to the sample were 26.1 °C, 23.1 °C, and 23.9 °C, respectively. When the sample was directly treated with plasma under this condition, it decomposed, as shown in the 60 min treatment condition in Fig. 4b (Supplementry Fig. 1). During the indirect plasma treatment using only the reactive species, approximately 96 mg/g of the newly formed compound was generated, and when the indirect plasma treatment was utilized while excluding the effects of the reactive species, approximately 19 mg/g of the compound was produced. As confirmed from these results, new compounds were also generated by the indirect plasma treatment while excluding RONS effects. The contents of isolated dimers A and B according to the plasma treatment methods used were quantified, and these results are summarized in Table 1.

**Table 1:** Contents of dimers A and B generated by plasma treatment method^a^.

Plasma treatment method	V_pp_ (kV)	t_treatment_ (min)	Dimer 3 (A) (mg/g)	Dimer 2 (B) (mg/g)
Direct	8	10	60.0 ± 2.1	111.8 ± 3.3
Indirect (only RONS)			44.5 ± 2.3	91.0 ± 2.8
Indirect (except for RONS)			n/d	n/d
Direct	9		n/d	n/d
Indirect (only RONS)			36.1 ± 0.9	60.3 ± 1.5
Indirect (except for RONS)			12.9 ± 0.5	5.8 ± 0.5

^a^Mean ± SD (n = 3), n/d: not detected.

**Figure 6:**
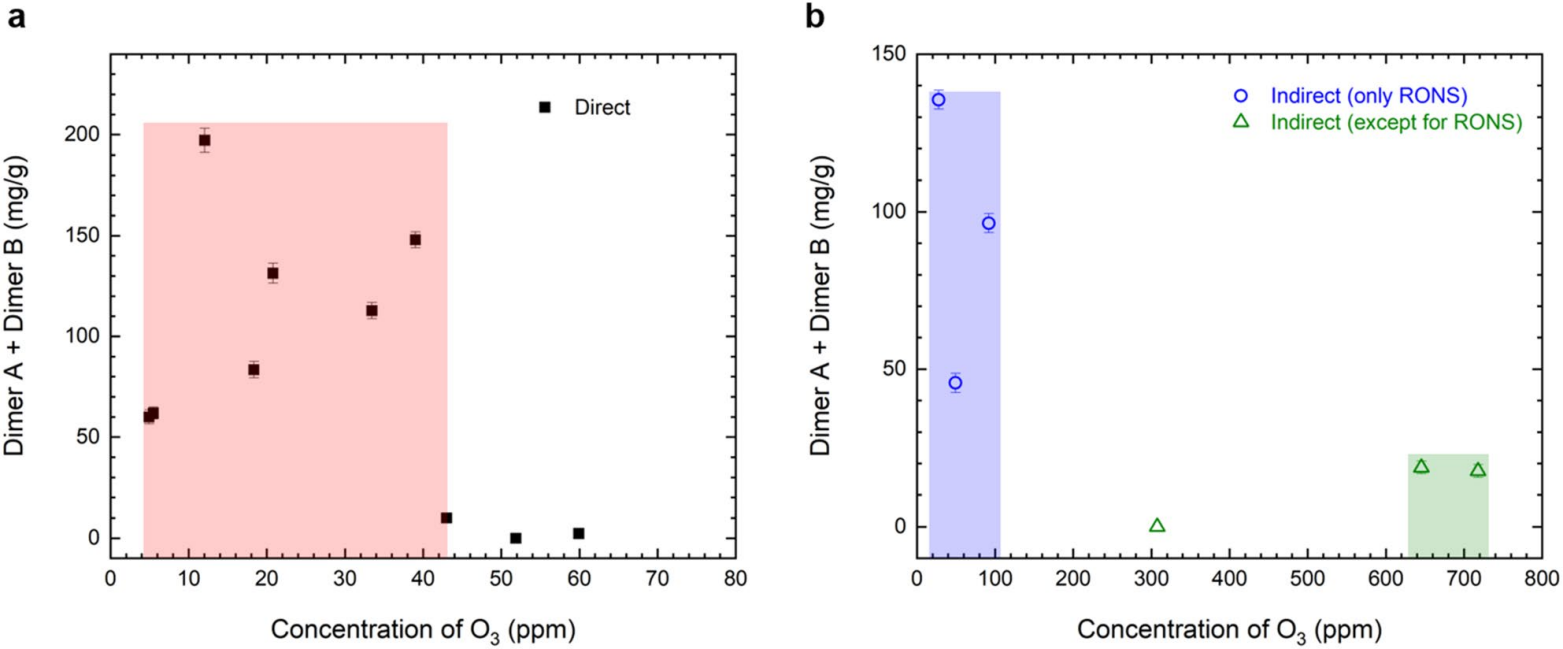
Contents of newly generated dimers A and B according to the plasma treatment method: (**a**) direct plasma treatment, and (**b**) indirect plasma treatment.

Figure 6 shows the contents of newly generated dimers A and B according to the ozone concentration as a major factor of the plasma treatment; here, the specific operating condition for regulated dimerization was observed for each plasma treatment. For the direct plasma treatment, Dimers A and B were produced at ozone concentrations below 43 ppm, but not at concentrations above 52 ppm as shown in Fig. 6a. However, even at a similar ozone concentration, the content of the newly generated compound showed a difference indicating that other factors also contribute to dimerization besides ozone. The ozone concentration range for efficient dimerization presented in Fig. 6 was demonstrated only under certain conditions and could change upon a change in the concentration and/or amount of the treated sample. For the plasma indirect treatment using only reactive species, new compounds were generated when the ozone concentration was 92 ppm or less, as shown in Fig. 6b. For the plasma indirect treatment excluding reactive species, dimerization was observed at a high concentration of 650 ppm or more of ozone, but the content of dimer A and B was found to be less than 20 mg/g in these cases. Dimers A and B were synthesized using only ozone generated by plasma and were also generated by plasma effects excluding reactive species. However, effective dimerization, i.e., low power consumption and a low ozone concentration, was realized when the sample was directly treated with plasma. Additional studies are required to find the ozone concentration operation window for regulated dimerization under various operating conditions, such as different concentrations and volumes of the sample and, rotation speeds of the stirrer, for instance, and to identify the plasma effect when excluding reactive species. This represents opportunities for further study.

### Mechanism of dimerization by plasma

**Figure 7:**
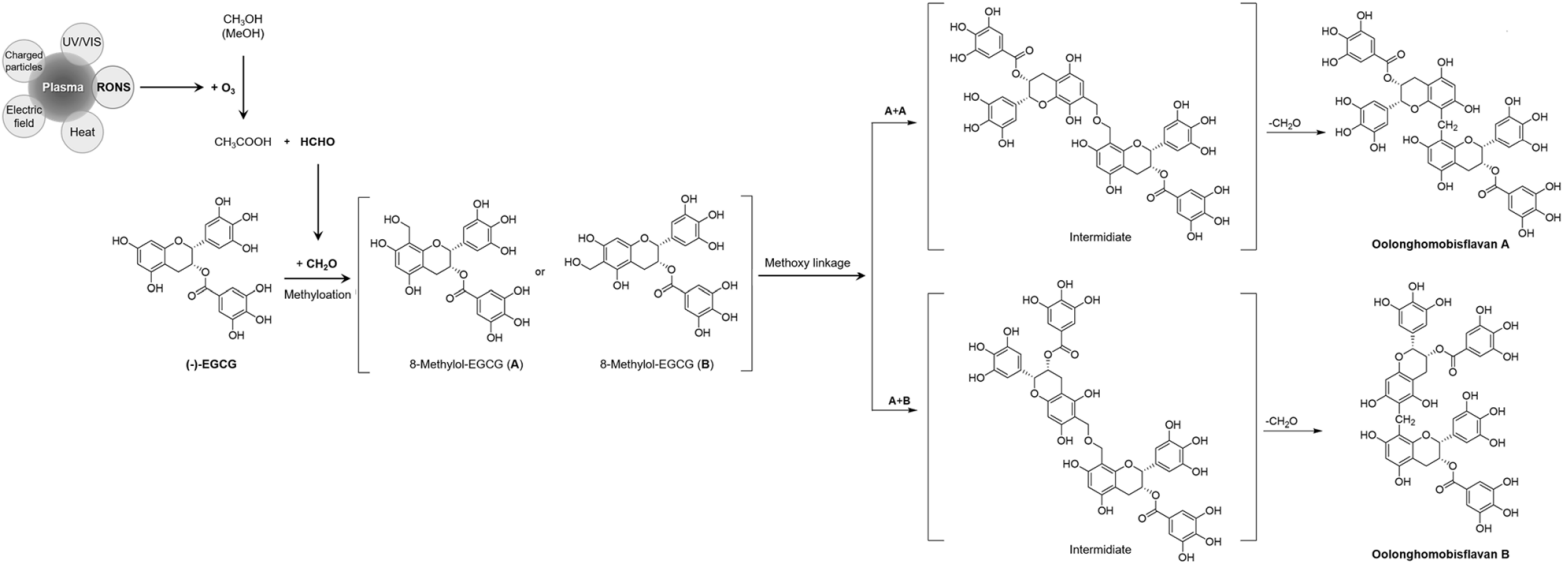
A plausible pathway of the dimerization of EGCG in a methanolic solution by the plasma treatment.

To understand the mechanism of EGCG dimerization by plasma, the literature on the relationship between EGCG and dimeric EGCG derivatives (oolonghomobisflavans A and B) was studied based on the present work. According to findings by Kiatgrajai et al.^22^, and Kiehlmann et al*.*^23^, flavan 3-ols analogue is generated as oligomers through a methylene-bridge by formaldehyde, and EGCG dimers **2** and **3** were synthesized and identified as oolonghomobisflavans A and B by Hahimoto et al*.*^24^ and Jeong et al*.*^19^. Regarding the finding that formaldehyde contributes to the dimerization of EGCG, further investigations into the relationship between methanol and formaldehyde were carried out, and it was confirmed by Rakovski et al*.* that ozone and methanol reacted to form formaldehyde^25^. We attempted to verify whether formaldehyde was generated in the plasma-treated sample here, and confirmed using GC–MS/MS (Thermo Scientific TRACE 1300 Series) that formaldehyde was indeed generated (Supplementry Fig. 2). Based on these results, a plausible pathway of the regulated dimerization of EGCG in a methanolic solution by a plasma treatment was deduced as shown in Fig. 7. The mechanism of dimerization by plasma proceeds as follows: ozone generated by plasma reacts with methanol to form formaldehyde, after which EGCG is generated as oolonghomobisflavans A and B through a methylene-bridge stemming from the formaldehyde reaction.

## Conclusion

We investigated the major factors of plasma to understand the effects of plasma on regulated dimerization of EGCG by varying the plasma treatment method. The influence of the plasma treatment was evaluated based on the contents of dimers A (3) and B (2), which were compounds that had newly formed, and samples were treated by a direct plasma treatment, an indirect plasma treatment using only reactive species, and an indirect plasma treatment excluding reactive species. Under identical operating conditions, the content of the main compounds produced by direct treatment amounted to 172 mg/g, with the indirect treatment with only reactive species producing approximately 136 mg/g of the compounds. With regard to indirect treatment excluding reactive species, dimers A and B were not generated under identical conditions. However, new major products amounting to approximately 19 mg/g were generated under higher applied voltage conditions, during which all samples decomposed when directly treated with plasma. These results indicate that ozone is the major factor from the plasma treatment and there is an ozone concentration operation window that depends on the operating conditions and treatment method used for efficient dimerization. Based on these results, a plausible pathway of dimerization was deduced. Ozone from plasma reacts with methanol to form formaldehyde, with oolonghomobisflavans A and B then synthesized through a methylene-bridge by formaldehyde. We believe that the present results will be useful information and or a valuable reference for those engaged in research on and applications of treatments of natural products using plasma.

## Supplementary Information

Supplementary Information.

## Data Availability

The datasets generated during and/or analyzed during the current study are available from the corresponding.
