# Supplementary material for: Mechanism of (−)-epigallocatechin gallate (EGCG) dimerization by low-temperature plasma
Source: Sci Rep. 2022 Sep 13;12:15396. doi: 10.1038/s41598-022-19806-0 (PMC9470551; doi:10.1038/s41598-022-19806-0)
Supplement: Supplementary file 1 — Supplementary Information. [file 41598_2022_19806_MOESM1_ESM.pdf]

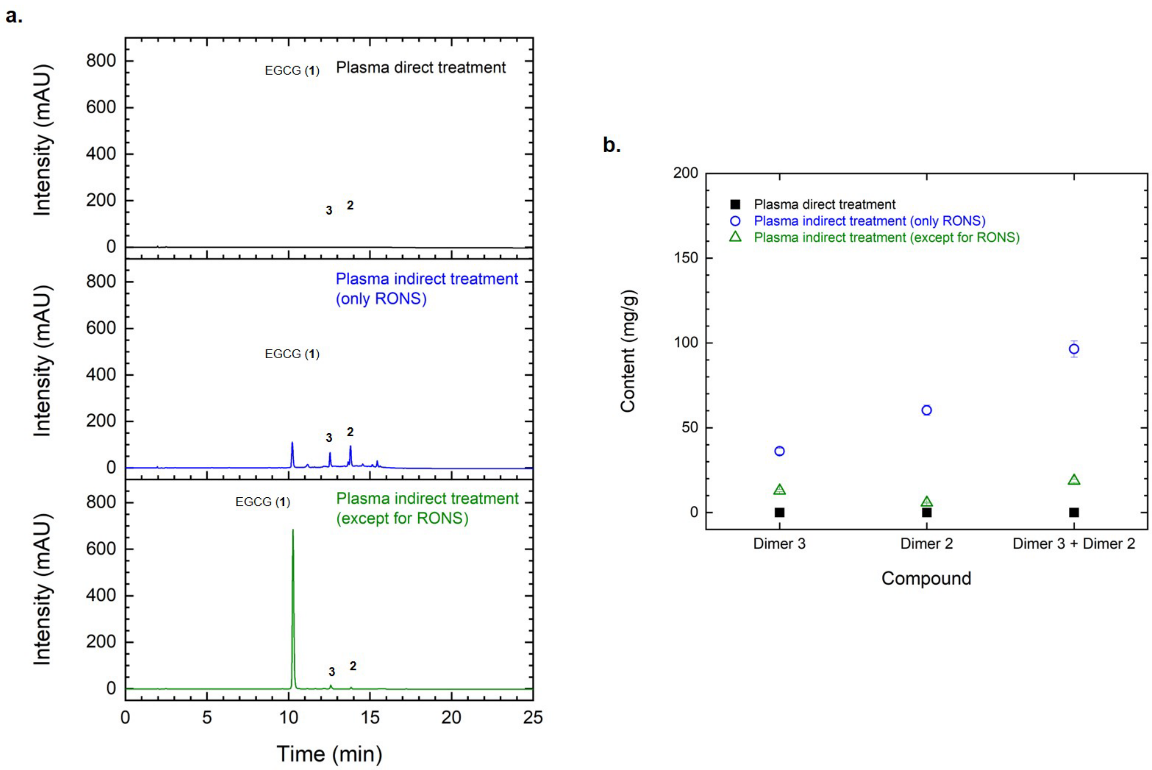

Supplementary Figure 1. (a) HPLC chromatograms of samples according to the plasma treatment conditions using a sine waveform with a peak-to-peak approximately 9 kV and frequency of 2.5 kHz, and (b) contents of individual components generated by the plasma treatment

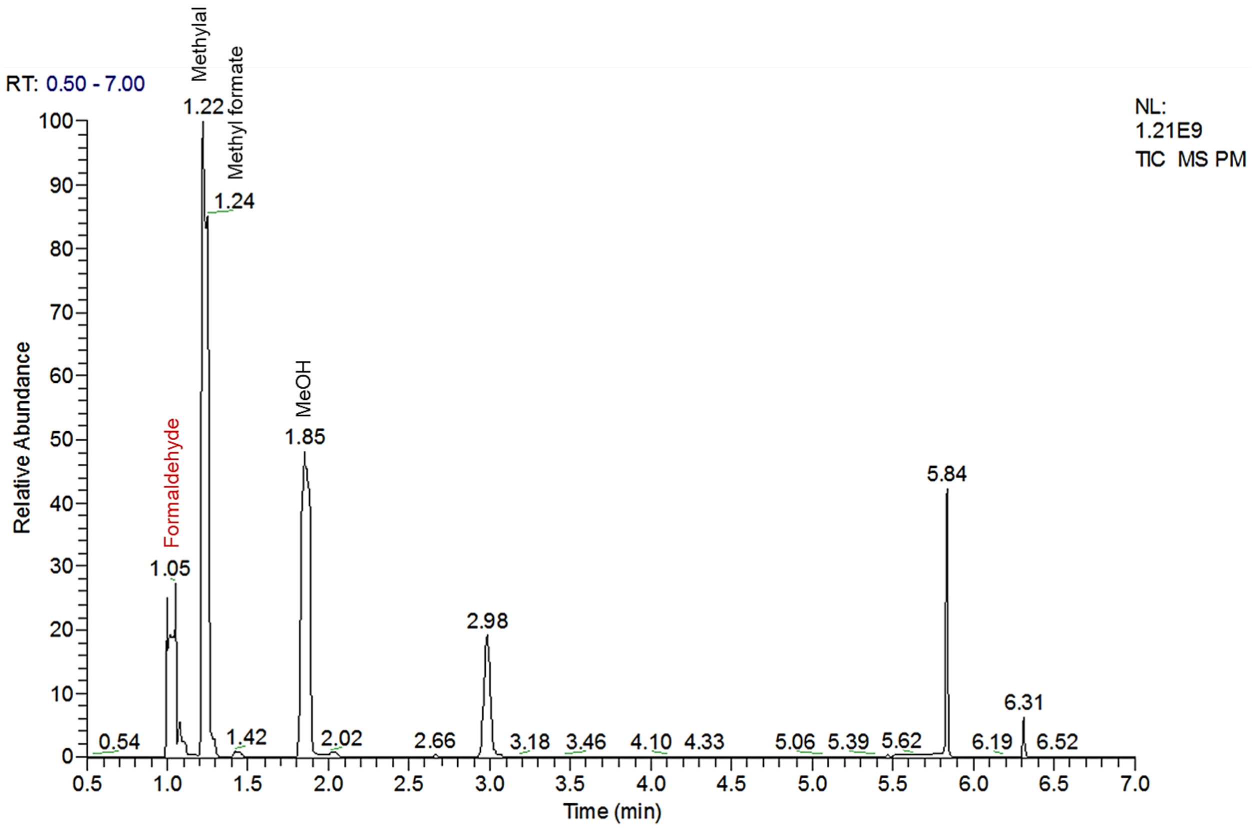

Supplementary Figure 2. GC-MS/MS chromatogram of plasma-treated samples
